# The Effect of Conflicting Public Health Guidance on Smokers’ and Vapers’ E-cigarette Harm Perceptions

**DOI:** 10.1093/ntr/ntac163

**Published:** 2022-07-06

**Authors:** Madeleine R E Svenson, Tom P Freeman, Olivia M Maynard

**Affiliations:** Department of Psychology, Addiction and Mental Health Group (AIM), University of Bath, Bath, UK; Department of Psychology, Addiction and Mental Health Group (AIM), University of Bath, Bath, UK; MRC Integrative Epidemiology Unit (IEU), School of Psychological Science, University of Bristol, Bristol, BS81TU, UK

## Abstract

**Background:**

E-cigarettes are increasingly being viewed, incorrectly, as more harmful than cigarettes. This may discourage smokers from switching to e-cigarettes. One potential explanation for these increasingly harmful attitudes is conflicting information presented in the media and online, and from public health bodies.

**Aims and Methods:**

In this prospectively registered online study, we aimed to examine the impact of conflicting public health information on smokers’ and vapers’ e-cigarette harm perceptions. Daily UK smokers who do not vape (*n* = 334) and daily UK vapers (*n* = 368) were randomized to receive either: (1) a consistent harm reduction statement from two different public health bodies (Harm Reduction), (2) a consistent negative statement about e-cigarette harms from two different public health bodies (Negative), (3) a harm reduction statement from one public health body and a negative statement from another (Conflict), and (4) a statement of the risks of smoking followed by a harm reduction statement from one public health body and a negative statement from another (Smoking Risk + Conflict). Participants then answered questions regarding their perceptions of e-cigarette harm.

**Results:**

The Negative condition had the highest e-cigarette harm perceptions, significantly higher than the Smoking Risk + Conflict condition (MD = 5.4, SE = 1.8, *p* < .016, *d* = 0.3 [CI 0.73 to 10.04]), which did not differ from the Conflict condition (MD = 1.5, SE = 1.8, *p* = .836, *d* = 0.1 [CI −3.14 to 6.17]). The Conflict condition differed from the Harm Reduction condition, where harm perceptions were lowest (MD = 5.4, SE = 1.8, *p* = .016, *d* = 0.3 [CI 0.74 to 10.07]).

**Conclusions:**

These findings are the first to demonstrate that, compared to harm reduction information, conflicting information increases e-cigarette harm perceptions amongst vapers, and smokers who do not vape.

**Implications:**

This research provides the first empirical evidence that conflicting information increases smokers’ and vapers’ e-cigarette harm perceptions, compared to harm reduction information. This may have a meaningful impact on public health as e-cigarette harm perceptions can influence subsequent smoking and vaping behavior. Conflicting information may dissuade smokers, who have the most to gain from accurate e-cigarette harm perceptions, from switching to e-cigarettes. These findings indicate that public health communications that are consensus-based can lower harm perceptions of e-cigarettes, and have the potential to reduce morbidity and mortality attributable to tobacco smoking.

## Introduction

In the United Kingdom, only 38% of smokers accurately believe that e-cigarettes are less harmful than cigarettes and beliefs that e-cigarettes are as or more harmful than cigarettes are on the rise.^1^ These misperceptions are a barrier to e-cigarette use among current smokers.^2^

The concept of e-cigarettes as a harm reduction tool has divided the public health community,^3^ leading to different regulatory approaches being adopted, with some countries, such as Australia and India, prohibiting the sale of nicotine-containing e-cigarettes.^4,5^ In England, most public health bodies concur that e-cigarettes are a harm reduction tool.^6^ In contrast, the World Health Organization (WHO) recommends against vaping on the basis of its’ potential harms, classifying e-cigarettes as harmful.^7^ Their stance has been strongly criticized by public health experts for perpetuating e-cigarette misperceptions and ignoring the potential of e-cigarettes as an effective smoking cessation aid.^8^ Exposure to this kind of conflicting information may increase e-cigarette harm perceptions over time,^9–11^ although having experience of vaping may mitigate this effect. Indeed, for never vapers, but not current vapers, reading conflicting as opposed to positive headlines reduced endorsement of e-cigarette benefits.^12^ Furthermore, conflicting information on e-cigarette packaging increased ambiguity among nonsmokers, but not smokers.^13^

The impact of experimental exposure to conflicting e-cigarette information on e-cigarette harm perceptions has received little empirical attention, and that which has been conducted has not focused on smokers.^12^ The current study, therefore, advances previous research by exploring the impact of conflicting public health information on smokers’ and vapers’ e-cigarette harm perceptions. This study also explored whether more accurate e-cigarette harm perceptions can be encouraged through the inclusion of a statement of the risks of smoking (thereby providing a high baseline risk) alongside conflicting information about e-cigarettes. Participants were randomized to one of the following conditions: (1) a consistent harm reduction statement from two different public health bodies (Harm Reduction), (2) a consistent negative statement about e-cigarette harms from two different public health bodies (Negative), (3) a harm reduction statement from one public health body and a negative statement from another (Conflict), and (4) a statement of the risks of smoking followed by a harm reduction statement from one public health body and a negative statement from another (Smoking Risk + Conflict). We hypothesized that:

- Hypothesis 1: E-cigarettes will be perceived as most harmful in the Negative condition, being perceived as progressively less harmful in the Conflict condition, the Smoking Risk + Conflict condition, and finally the Harm Reduction condition where e-cigarettes will be perceived as least harmful.
- Hypothesis 2: Across all study conditions, smokers who do not vape (smoker-non vapers), will have higher e-cigarette harm perceptions than vapers.
- Hypothesis 3: Vapers’ harm perceptions will be less impacted by the information communicated than smoker-non vapers.

## Method

### Participants

Participants (*n* = 714) were recruited via Prolific Academic, an online crowd-sourcing platform (https://www.prolific.co/). The survey was only available to members of Prolific Academic who had previously reported that they were: 18 years or older; UK residents; either daily smokers, who had vaped less than 20 times in their lifetime; or those who vape daily and did not specify their smoking status. An a priori power calculation using G*Power indicated that 640 participants were required to observe a small effect size (*f* = 0.14) with 95% power at an alpha level of 5%, for Analysis of Variance (ANOVA) interaction effects between group and condition.^14^ We planned to recruit 716 participants to account for dropout because of participants failing the attention check, the manipulation check, or not meeting the inclusion criteria. Ethics approval was obtained from the School of Psychological Science Human Research Ethics Committee at the University of Bristol (Approval Code: 102882) and the Psychology Research Ethics Committee at the University of Bath (Approval Code: UG20-007).

### Design

The study used an online between-subjects experimental design, with four conditions. Participants were either smoker-nonvapers (daily smokers who have vaped fewer than 20 times in their lifetime) or vapers (daily vapers whose smoking status is not specified). Participants were randomized to one of the four conditions where they read short statements about e-cigarettes (Harm Reduction; Conflict + Smoking Risk; Conflict; Negative; see Table 1 for full details of the conditions including the specific wording used). The randomization was designed to ensure there were an equal number of males and females, and an equal number of smoker-nonvapers and vapers under each condition. In both conflict conditions, the order of presentation of the harm reduction and negative statement was counterbalanced. The outcome measure was e-cigarette harm perceptions. Prior to data collection, the study protocol and hypotheses were published on the Open Science Framework (OSF; https://osf.io/5yhuk/).

**Table 1.** Experimental Conditions

Negative
Public health organization 1 reports that the risk of e-cigarettes depends on a range of factors, but e-cigarettes pose clear health risks and are by no means safe.
Public health organization 2 also reports that the risk of e-cigarettes depends on a range of factors, but e-cigarettes pose clear health risks and are by no means safe.
Conflict
Public health organization 1 reports that while e-cigarettes are not risk free, they carry a fraction of the risk of cigarettes.
Public health organization 2 reports that the risk of e-cigarettes depends on a range of factors, but e-cigarettes pose clear health risks and are by no means safe.
Smoking risk + Conflict
Smoking traditional cigarettes is uniquely harmful. Tobacco smoking kills two in three-lifetime users. Smokers can expect to die 10 years earlier than nonsmokers.
Public health organization 1 reports that while e-cigarettes are not risk free, they carry a fraction of the risk of cigarettes.
Public health organization 2 reports that the risk of e-cigarettes depends on a range of factors, but e-cigarettes pose clear health risks and are by no means safe.
Harm reduction
Public health organization 1 reports that while e-cigarettes are not risk free, they carry a fraction of the risk of cigarettes.
Public health organization 2 also reports that while e-cigarettes are not risk free, they carry a fraction of the risk of cigarettes.

### Measures and Materials

#### Stimuli

Real-world public health messages were used as the stimuli. The public health bodies were not specifically named; instead, the generic terms “public health organization” 1 or 2 were used. The Harm Reduction statement was based upon the current Public Health England (PHE) advice (https://publichealthmatters.blog.gov.uk/2019/10/29/vaping-and-lung-disease-in-the-us-phes-advice/). PHE states that “E-cigarettes are not risk free but are far less harmful than cigarettes” with “evidence still showing vaping carries a small fraction of the risk of smoking.”

The Negative statement was formed from a tweet by the WHO (https://twitter.com/who/status/1219618083645595650?s=21) reading “Q: Are e-cigarettes more dangerous than regular cigarettes? A: This depends on a range of factors, including the amount of nicotine and other toxicants in heated liquids, but we know that e-cigarettes pose clear health risks and are by no means safe.” The middle clause was removed to avoid technical language. The smoking risk statement was taken from a BBC news article reporting on a study^15^ (https://www.bbc.co.uk/news/health-31600118) reading “tobacco kills two in three smokers.” See Table 1 for the excerpts as presented to the participants.

### Measures

#### E-cigarette Harm Perceptions

This five-item e-cigarette harm perception measure evaluates how harmful participants believe e-cigarettes are as a standalone product, and in comparison to cigarettes. It quantifies participants’ level of agreement with the statements “E-cigarettes are harmful,” “E-cigarettes are less harmful than combustible cigarettes”; “E-cigarettes are a helpful tool for people who want to quit smoking”; “There is convincing scientific evidence that e-cigarettes are safe” and “There is convincing scientific evidence that e-cigarettes are safer than smoking.”^15^ The scale has high internal consistency (α = 0.83). Responses are measured on 101-point scales, with 0 representing “strongly disagree” and 100 representing *“*strongly agree.*”* We used a 101-point scale, which, as a continuous variable, was appropriate for the analyses we conducted. Four items are reverse coded so that higher scores indicate agreater endorsement of e-cigarette harm and an overall mean of the five items is taken.

#### Smoking and Vaping

Participants answered questions about their frequency of e-cigarette and cigarette use, the number of cigarettes they smoked per day, week or month as appropriate, and how many times they have made serious quit attempts. Participants who smoked also completed the Quitting Smoking Contemplation Ladder (QSCL)^16^ and the Fagerström test for Cigarette Dependence.^17^ Participants who vaped indicated their length of use and completed the QSCL that we adapted for vaping (words smoking and cigarette replaced with vaping and e-cigarette respectively) and the e-cigarette Fagerström Test for Nicotine Dependence.^18^

#### Demographic Questions

Demographic information included the highest level of academic qualification obtained, student status, occupation, gender, age, and UK residence.

#### Attention Check

At the end of the e-cigarette harm perception measures, participants were presented with an attention check instructing them to “Respond strongly agree (100) to this question.”

#### Manipulation Check

After completing the attention check, participants indicated, “based only off the information you read and not your personal opinions, would you say that public health bodies’ view of e-cigarettes is; e-cigarettes are less harmful than cigarettes; e-cigarettes are harmful; conflicted about the harms of e-cigarettes.”

### Procedure

The experiment, conducted via Qualtrics, took on average 5 minutes 46 seconds to complete. Participants read an information sheet and then provided informed consent. Participants were asked about their frequency of smoking and vaping to confirm their eligibility. If participants were ineligible, they were directed to the end of the survey and not reimbursed. Eligible participants were randomized to one of the four conditions. After reading the assigned e-cigarette condition information, participants answered the harm perception questions and completed an attention check and manipulation check. Next, participants answered questions about their smoking and vaping behavior based on whether they were smokers, vapers, or dual users. Participants then answered demographic questions. Upon completion, participants were debriefed and reimbursed £1 through Prolific Academic.

### Data Analysis

Statistical analysis was conducted in SPSS version 26. Participants who failed the attention check or did not meet the inclusion criteria were removed from the analysis. A two-way (2 × 4) ANOVA was planned to analyze differences in mean harm perception scores between group (smoker-nonvaper; vaper) and condition (Harm Reduction; Smoking Risk + Conflict; Conflict; Negative). Differences in harm perceptions between conditions were compared using Tukey’s honestly significant difference (HSD) post hoc comparisons. Levene’s test was used to assess homogeneity of variance. Shapiro–Wilks tests and visual inspection of Q–Q plots were used to assess normality. There were minor deviations from normality. Despite this, ANOVA and Tukey’s HSD post hoc comparisons were run as they are robust to violations of normality.^19,20^ Effect sizes were classified according to Cohen’s classification (*d* ≥ 0.2 small effect, *d* ≥ 0.5 medium effect, *d* ≥ 0.8 large effect).^21^ Differences in demographic factors between smoker-nonvapers and vapers were explored using *χ*^2^, always tests for categorical variables, and Mann–Whitney tests for age as it was not normally distributed. *t*-Tests were used to explore whether the counterbalancing of conditions was effective. The counterbalanced conditions did not differ in harm perceptions; therefore, we combined these conditions for all analyses.

## Results

Data are available at the University of Bristol data repository, data.bris, at https://doi.org/10.5523/bris.acd8vbd07q8f2u3rw3unq25nn.

### Participants

A higher (*n* = 278) than expected (*n* = 76) number of participants failed the manipulation check remaining (*n* = 424). Participants in the conflicting conditions disproportionally failed the manipulation check (Harm Reduction *n* = 21; Negative *n* = 51; Conflict *n* = 107; Smoking Risk + Conflict *n* = 99). Of the participants in the Conflict and Smoking Risk + Conflict condition that failed the manipulation check (*n* = 206), 78% (*n* = 160) indicated e-cigarette are less harmful than cigarettes, whilst only 22% (*n* = 46) failed because they indicated that e-cigarettes are harmful, suggesting that a positivity bias in responding. Thus, deviating from the preregistered analysis plan, participants who failed the attention check were not excluded. Analyses excluding participants thatwho failed the manipulation check were conducted as a sensitivity analysis, which is described below and reported in full in Supplementary Materials.

A total of 714 participants completed the experiment. Eight participants were excluded for not being UK residents and four participants were excluded for failing the attention check, leaving a total of 702 participants (Harm Reduction *n* = 174, Negative *n* = 176, Conflict *n* = 176, Smoking Risk + Conflict *n* = 176).

The sample comprised smoker-nonvapers (*n* = 334; 49% female, mean age 38.7 [SD=12.2]) and vapers (*n* = 368; 50% female, mean age 38.6 [SD = 11.9]). Of the vapers, 41% (*n* = 149) smoked daily, 9% (*n* = 33) smoked weekly, 6% (*n* = 21) smoked monthly, and 45% (*n* = 165) smoked less than monthly or not at all. Of the vapers that smoked less than monthly or not at all, 93% (*n* = 154) were former smokers (and of these, 94% were previously daily smokers), suggesting that they started vaping to quit smoking. Only 7% (*n* = 11) of the vapers had never previously smoked.

As shown in Supplementary Table 1, smokers had smoked between 7.5 (SD = 6.2) and 19.8 (SD = 9.2) cigarettes per day on average over the past 2 months, whilst vapers who smoked daily, weekly or monthly, smoked between 5.5 (SD = 18.6) cigarettes per day over the past 2 months. As shown in Supplementary Table 2, the largest proportion of vapers (55%) had been vaping for over 2 years.

Demographic characteristics (i.e. age, education, and occupation) were well balanced between the smoker-nonvapers and vapers (see Supplementary Table 1 for additional participant characteristics).

### Primary Analysis

#### E-Cigarette Harm Perceptions

**Figure 1.**
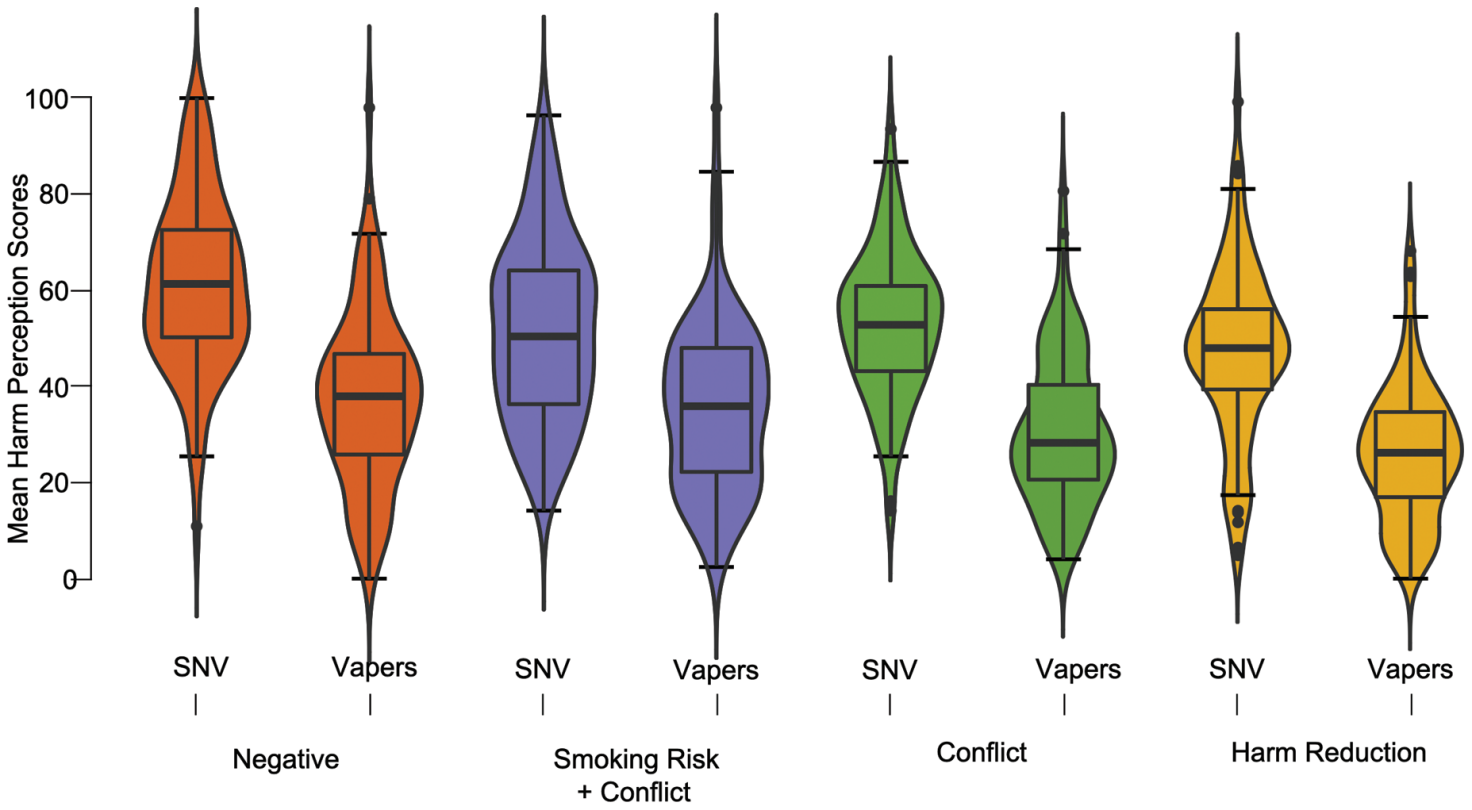
Violin plots showing the distribution of e-cigarette harm perceptions. The central horizontal line represents the median, the outside of the box represents the inter-quartile range, the outer horizontal lines indicate the 95% confidence intervals. Outliers are presented as dots. SNV = smoker-nonvaper.

There was a main effect of condition on mean e-cigarette harm perception scores (*F*(3,694) = 16.47, *p* < .001, η^2^ = 0.07). As illustrated in Figure 1, those in the Negative condition had the highest harm perceptions (*M* = 49.4, SD = 21.58), followed by those in the Smoking Risk + Conflict condition (*M* = 43.6, SD = 19.80), and the Conflict condition (*M* = 41.9, SD = 19.02; no evidence for a meaningful difference in scores between these two groups), with lowest harm perceptions in the Harm Reduction condition (*M* = 36.7, SD = 19.27). Post hoc comparisons between conditions are shown in Table 2.

**Table 2.** Tukey’s HSD Comparison of Mean Harm Perception Scores by Condition

Condition 1	Condition 2	MD (Condition 1–Condition 2)	SE	*P*	*d*
Negative	Smoking Risk + Conflict	5.4	1.8	.016	0.3
Negative	Conflict	6.9	1.8	.001	0.3
Negative	Harm Reduction	12.3	1.8	<.001	0.6
Smoking Risk + Conflict	Conflict	1.5	1.8	.836	0.1
Smoking Risk + Conflict	Harm Reduction	6.9	1.8	.001	0.4
Conflict	Harm Reduction	5.4	1.8	.016	0.3

MD = mean difference; SE = standard error; *d* = Cohen’s *d* effect size; *p*-values have been corrected for multiple comparisons.

### Smoking and Vaping Status

There was a main effect of group on mean e-cigarette harm perception scores, *F*(1,694) = 265.74, *p* < .001, η^2^ = 0.28) and this effect was in the expected direction, with smoker-nonvapers (*M* = 53.4, SD = 18.14) having higher harm perceptions than vapers (*M* = 32.5, SD = 16.94).

### Impact of Condition by Smoking and Vaping Status

As shown in Figure 1, there was no evidence for an interaction between experimental condition and group on e-cigarette harm perceptions scores (*F*(3,694) = 1.57, *p* = .196, η^2^ = 0.01).

### Sensitivity Analysis

The sensitivity analysis (excluding those participants who failed the manipulation check) produced the same pattern of results as the main analysis (see Supplementary Tables 4–7). The effect sizes for all comparisons were similar to the main analysis (Cohen’s *d* within 0.2 of each other). One difference was that there was evidence that the Harm Reduction condition differed from the Smoking Risk + Conflict in the main analysis but not the sensitivity analysis, although the magnitude of effect was similar across both analyses. The results of the sensitivity analysis should be interpreted with caution because of the biased operation of the manipulation check across conditions and because the sensitivity analysis is underpowered relative to the planned sample size for this study.

## Discussion

This experimental study demonstrates that brief exposure to either negative or conflicting information from public health bodies heightens both smokers’ and vapers’ e-cigarette harm perceptions compared to harm reduction information. This provides empirical support to the purported link between the prevalence and proliferation of conflicting information in the media, and heightened e-cigarette harm perceptions among UK smokers.^9–11^ Increased e-cigarette harm perceptions are problematic as they may discourage e-cigarette use among smokers.^2^

We observed higher e-cigarette harm perceptions among smokers compared to vapers, which supports an established body of research.^9^ However, contrary to our expectations, both smokers and vapers responded to the e-cigarette information in a similar manner, such that negative and conflicting information also increased vapers’ harm perceptions, albeit to a lesser extent than amongst the smokers. This suggests that conflicting information can also negatively impact those with experience of vaping. The implications of these findings are unclear, although research among dual users suggests that heightened e-cigarette harm perception may encourage the maintenance of dual use.^22^ This finding is a departure from previous research which has found that conflicting information increases non-vapers’, but not vapers’ e-cigarette harm perceptions,^12^ and does not affect smokers’ harm perceptions.^13^ This finding is potentially a result of both our vapers and the nonvapers being unsure of the scientific evidence and consequently responding similarly to the information presented. Indeed, knowledge levels and issue involvement between smokers and vapers may have been more similar than expected. Whilst the smokers were nonvapers, they may have still tried e-cigarettes, as have 60% of smokers in the United Kingdom.^23^

We did not find that harm perceptions were lowered when smoking risk information was included alongside conflicting information. These findings are contrary to previous research, which indicates that the communication of baseline risk results in more accurate risk perceptions.^24^ This may be because harm perceptions are formed in reference to an individual’s own personal comparison starting point (i.e. anchor).^13^ As the risks of smoking are well known, and the majority of the sample either smoked, or smoked and vaped, their reference point may already have been the risk of smoking. Thus, the introduction of a smoking risk statement may not have changed how they formed their harm perceptions.

Our findings suggest that public health bodies should communicate the safety of e-cigarettes in consensus with other public health bodies to reduce harm perceptions. This is feasible in light of the growing body of evidence, suggesting that e-cigarettes are an effective smoking cessation tool and therefore have the potential to reduce smoking-associated morbidity and mortality.^25^ Additionally, public health bodies may wish to proactively challenge negative information by countering it with harm reduction information, as we find that negative information is more harmful than conflicting information. However, when public health bodies are already in conflict, it is not necessarily advantageous to reiterate the harms of smoking. These communication methods need to be evaluated among people who do not smoke to ensure that vaping is not promoted amongst this group.

Key strengths of this study include high statistical power through a large sample size ensuring that small effects, which typify communications research, could be detected and minimizing the risk of false-positive findings.^26^ Moreover, the randomized design enabled causal inference that the changes in harm perceptions are a result of the manipulation. Preregistration of our hypotheses and analysis plans also increase the robustness of the findings.

There are however limitations to our research. First, the manipulation check was ineffective as there was evidence that participants’ previous attitudes likely influenced whether they construed conflicting information as harm reduction or negative. Moreover, there was a positivity bias whereby participants incorrectly indicated conflicting information as harm reduction information. This may be because the harm reduction condition was phrased as relative risk: “e-cigarettes are less harmful than cigarettes” whilst the negative condition was framed in terms of absolute risk: “e-cigarettes are harmful.” This failure of the manipulation check does not seem to have impacted the results as the sensitivity analysis produced the same pattern of results when participants who failed the manipulation check were excluded. Second, our operationalization of “conflict” has both strengths and potential limitations. The public health bodies were not named which strengthens our inferences that the information within the extract, rather than information about the source, was responsible for the observed effects, although it is important to note that the impact of conflicting information is influenced by the dissemination channel.^27^ Furthermore, our findings relate to conflicting information operationalized as two health-related statements that are logically inconsistent. However, the findings may not apply to alternative conceptualizations of conflicting information such as one behavior, vaping, producing two or more outcomes.^28^ For example, e-cigarettes benefitting smokers’ lung health whilst also having unknown long-term health risks. Third, given this was an online study, we relied on self-reported smoking and vaping behavior rather than biochemical validation of these important characteristics. Finally, future research should seek to establish to what extent the changes in harm perceptions outlined here translate into meaningful real-world effects on subsequent smoking and vaping behavior.

## Conclusion

This study highlights that conflicting information, compared to harm reduction information, increases harm perceptions amongst both vapers and smokers, a population whose health would benefit most from switching to e-cigarettes. This study provides support for the hypothesized role of conflicting information, alongside negative information, in rising harm perceptions of e-cigarettes. The findings may have important public health implications as, although the effects are small, at a population level, conflicting information could meaningfully influence downstream smoking and vaping behavior, exacerbating the smoking related public health burden. Public health bodies should focus on consensus-based harm reduction messaging to lower smokers’ and vapers’ harm perceptions. Moreover, public health bodies should counter negative information about e-cigarettes promoted by other public health organizations.

## Supplementary Material

A Contributorship Form detailing each author’s specific involvement with this content, as well as any supplementary data, are available online at https://academic.oup.com/ntr.

## Supplementary Material

ntac163_suppl_Supplementary_Material

ntac163_suppl_Supplementary_Taxonomy-form

## Data Availability

Data are available at the University of Bristol data repository, data.bris, at https://doi.org/10.5523/bris.acd8vbd07q8f2u3rw3unq25nn.
