# Supplementary material for: The Effect of Conflicting Public Health Guidance on Smokers’ and Vapers’ E-cigarette Harm Perceptions
Source: Nicotine Tob Res. 2022 Jul 6;24(12):1945–50. doi: 10.1093/ntr/ntac163 (PMC9653072; doi:10.1093/ntr/ntac163)
Supplement: ntac163_suppl_Supplementary_Material [file ntac163_suppl_supplementary_material.docx]

**The effect of conflicting public health guidance on smokers and vapers’ e-cigarette harm perceptions**

Supplementary Tables 1 – 3 provide further demographic information about participants

Supplementary Tables 4 – 7 provide details of the sensitivity analyses where participants who failed the manipulation check are removed and the main analyses are re-run.

**Supplementary Table 1.** *Mean and standard deviations (SD) for smoking behaviours*

| Smoking Behaviour | Smoker-Non-Vapers  (n = 334) | | Vapers * | |
| --- | --- | --- | --- | --- |
|  |  | | Who currently smoke cigarettes daily, weekly or monthly  (n = 203) | |
|  | *Mean* | *SD* | *Mean* | *SD* |
| Minimum number of cigarettes smoked per day over last 2 months | 7.5 | 6.2 | 5.5 | 18.6 |
| Maximum number of cigarettes smoked per day over last 2 months | 19.8 | 9.2 | 14.9 | 29.5 |
| Number of smoking quit attempts | 2.4 | 3.6 | 3.7 | 4.6 |
|  |  |  | Who currently smoke cigarettes daily or weekly  (n = 182) | |
| Quitting Smoking Contemplation Ladder | 5.2 | 1.8 | 6.2 | 2.0 |
| Nicotine Dependence (FTND) + | 3.9 | 2.4 | 3.0 | 2.3 |

*Note.*

*+ Nicotine dependence only measures dependence to nicotine delivered by smoking cigarettes*

** Data for vapers excludes 165 participants who smoke less than monthly or not at all (and therefore were not asked smoking behaviour questions).*

**Supplementary Table 2.** *Vaping behaviour*

| Vaping Behaviour | | Vapers (n = 368) |
| --- | --- | --- |
| Length of Vaping | |  |
|  | 1-6 months | 33 (9%) |
|  | 7-12 months | 56 (15%) |
|  | 13-24 months | 77 (21%) |
|  | More than two years | 202 (55%) |
| Nicotine Dependence (e-FTND) + | | 4.2 (SD ± 2.7) |
| Number of Vaping Quit Attempts | | 0.5 (SD 1.2) |
| Quitting Vaping Contemplation Ladder | | 4.0 (SD ± 1.8) |

*Note.*

*+Nicotine dependence only measures dependence to nicotine delivered via e-cigarettes*

*SD=Standard deviation.*

| **Supplementary Table 3.** *Education, student status and profession of chief income earner for all participants, smoker-non-vapers and the vaper groups* | | | |
| --- | --- | --- | --- |
| Participant Characteristics | Participants  (n=702) | Smoker-non-vapers  (n = 334) | Vapers  (n = 368) |
| *Education* |  |  |  |
| Higher Education or professional/vocational equivalents | 335 (48%) | 153 (46%) | 182 (50%) |
| A-levels or vocational level 3 or equivalents | 183 (26%) | 90 (27%) | 93 (25%) |
| GCSE/O-Level grade A*-C, or vocational level 2 or equivalents | 133 (19%) | 66 (20%) | 67 (18%) |
| GCSE/O Level grace C or below, or qualification at level 1 or below | 34 (5%) | 16 (5%) | 18 (5%) |
| Other qualifications level unknown | 3 (0.4%) | 1 (0.3%) | 2 (0.5%) |
| No qualifications | 14 (2%) | 8 (2%) | 6 (2%) |
|  |  |  |  |
| *Student status* |  |  |  |
| Students | 74 (10.5%) | 35 (10.5%) | 39 (10.6%) |
| Undergraduate | 55 (7.8%) | 27 (8%) | 28 (8%) |
| Postgraduate | 19 (2.7%) | 8 (2%) | 11 (3%) |
|  |  |  |  |
| *Profession of chief earner* |  |  |  |
| Higher managerial/ professional/ administrative | 48 (7%) | 20 (6%) | 28 (8%) |
| Intermediate managerial/ professional/ administrative | 169 (24%) | 67 (20%) | 102 (28%) |
| Supervisory or clerical/ junior managerial/ professional/ administrative | 215 (31%) | 103 (31%) | 112 (30%) |
| Student | 25 (4%) | 13 (4%) | 12 (3%) |
| Skilled manual worker | 105 (15%) | 54 (16%) | 51 (14%) |
| Semi or unskilled manual work | 61 (9%) | 35 (10%) | 26 (7%) |
| Casual worker – not in permanent employment | 4 (0.6%) | 2 (0.6%) | 2 (0.5%) |
| Housewife/ Homemaker | 11 (2%) | 2 (0.6%) | 9 (2%) |
| Retired and living on state pension | 6 (0.9%) | 3 (0.9%) | 3 (0.8%) |
| Unemployed or not working due to long-term sickness | 51 (7%) | 30 (9%) | 21 (6%) |
| Full-time carer of another household member | 7 (1%) | 5 (2%) | 2 (0.5%) |
| *Note: GCSEs/O-Levels are UK school exams taken age 14-16. A-Levels are UK school exams taken age 16-18.*  *Values represent number and percentage this represents within each group.* | | | |

**Supplementary Table 4****.** *Sensitivity analysis excluding those who failed the manipulation check: Mean harm perception scores for participants in each condition*

| Condition | *Mean harm perception score (standard error)* |
| --- | --- |
| Negative (n = 125) | 51.91(1.48) |
| Conflict (n = 69) | 43.03 (1.99) |
| Smoking Risk + Conflict (n = 77) | 40.54 (1.88) |
| Harm Reduction (n = 153) | 35.68 (1.34) |

**Supplementary Table 5****.** *Sensitivity analysis excluding those who failed the manipulation check: Mean harm perception scores for smoker-non-vapers and vapers.*

| Group | *Mean harm perception score (standard error)* |
| --- | --- |
| Smoker-Non-Vaper (n=214) | 53.64 (1.18) |
| Vaper (n=210) | 31.94 (1.21) |

| **Supplementary Table 6****.**  *Sensitivity analysis excluding those who failed the manipulation check: Summary of ANOVA results* | | | | | |
| --- | --- | --- | --- | --- | --- |
| ANOVA | df | df error | F | *p* | η^2^ |
| Condition | 3 | 416 | 22.59 | <.001 | 0.140 |
| Smoking & Vaping Status | 3 | 416 | 163.91 | <.001 | 0.283 |
| Interaction (Smoking & Vaping Status*Condition) | 3 | 416 | 0.41 | .745 | 0.003 |

**Supplementary Table 7.** *Sensitivity analysis excluding those who failed the manipulation check: Tukey’s HSD comparison of mean harm perceptions scores between conditions*

| Condition 1 | Condition 2 | MD (Condition 1-Condition 2) | SE | *p* | *d* |
| --- | --- | --- | --- | --- | --- |
| Negative | Harm Reduction | 16.2 | 2.0 | <.001 | 0.8 |
| Negative | Conflict | 8.3 | 2.5 | .050 | 0.4 |
| Negative | Smoking Risk + Conflict | 10.9 | 2.4 | <.001 | 0.5 |
| Smoking Risk + Conflict | Conflict | -2.6 | 2.7 | .770 | -0.1 |
| Smoking Risk + Conflict | Harm Reduction | 5.3 | 2.3 | .100 | 0.3 |
| Conflict | Harm reduction | 7.9 | 2.4 | .050 | 0.4 |

*Note. MD = mean differences, SE = standard error, d = Cohen’s d effect size*
